# GLP-1 receptor agonist increase retained gastric contents on EGD and same-day colonoscopy reduces this risk

**DOI:** 10.3389/fmed.2025.1638981

**Published:** 2025-09-10

**Authors:** Dirin Ukwade, Luis J. Hernandez, Zeel Modi, Hasan S. Raza, Michael Siegel, Dexter Nwachukwu, Paya Sarraf, Abdullah Memon, Marya Booth, Karen Cooper, Brian R. Boulay, Ece A. Mutlu

**Affiliations:** ^1^Department of Medicine, University of Illinois Chicago, Chicago, IL, United States; ^2^Division of Gastroenterology & Hepatology, University of Illinois Chicago, Chicago, IL, United States

**Keywords:** glucagon-like peptide 1 receptor agonist, endoscopy, retained gastric contents, GLP-1RA, upper endoscopy, esophagogastroduodenoscopy (EGD)

## Abstract

**Introduction:**

With the rise in glucagon-like peptide 1 receptor agonist (GLP-1RA) medication usage for Type 2 diabetes mellitus and weight loss, concerns have been raised regarding safety and primary aspiration risk when undergoing anesthesia procedures. Given the paucity of evidence, there is concern whether patients on GLP-1RA are at higher risk of retained gastric contents and subsequent adverse outcomes during routine esophagogastroduodenoscopy (EGD). This study aims to investigate whether patients on GLP-1RA are at higher risk of retained gastric contents during routine EGD.

**Methods:**

In this retrospective study, we examined 1,368 adult patients who underwent EGDs in the outpatient setting at a tertiary care center. A multivariable analysis was conducted to predict the presence of retained gastric contents on EGD, with the primary predictor being GLP-1RA use. Covariates thought to contribute to delayed gastric emptying were used as secondary predictors.

**Results:**

Retained gastric contents were seen in 18 out of 128 cases in the GLP-1RA users (14.1%), which was statistically significant when compared to 45 out of the 1,156 non-users (3.8%) (*p* < 0.001, LR 18.323). There was no significant increase in adverse outcomes associated with this finding. GLP-1RA use (*p* < 0.001, OR = 5.4), history of gastroparesis (*p* < 0.001, OR = 4.55), chronic kidney disease (*p* = 0.036, OR = 3.47) and hemiplegia (*p* = 0.048, OR = 2.9) increased risk of retained gastric contents. In contrast, bowel prep (*p* = < 0.001, OR = 0.157) for same day lower GI procedures decreased risks.

**Conclusion:**

Our results show an increase in retained gastric contents in GLP-1RA users undergoing EGD. Other mitigating factors and whether the increase results in aspiration complications should be further studied.

## Introduction

The implementation of glucagon-like peptide 1 receptor agonist (GLP-1RA) medications has increased significantly over the past few years due to demonstrated efficacy with both weight loss and improvement in outcomes related to type 2 diabetes mellitus (T2DM). Glucagon-like peptide 1 is a peptide hormone that contributes to post-prandial glycemic control through a mechanism of action that is thought to affect the motility of the stomach (1). As a result, there have been concerns raised about the safety, primarily from an aspiration risk standpoint, in patients taking GLP-1RA who are also undergoing esophagogastroduodenoscopy (EGD). A recent multi-society statement by the American Gastroenterological Association (AGA), American Association for the Study of Liver Diseases (AASLD), American College of Gastroenterology (ACG), American Society for Gastrointestinal Endoscopy (ASGE), and the North American Society for Pediatric Gastroenterology, Hepatology & Nutrition (NASPGHAN) recommended exercising best practices to decide whether GLP-1RA should be held prior to performing EGD in the setting of insufficient data to support stopping these medications. Given the paucity of data surrounding outcomes in this population, we wanted to investigate if there is indeed a risk of retained gastric contents and subsequent adverse outcomes.

GLP-1RA were first developed with the intent to manage T2DM due to their ability to lower blood glucose levels. Subsequently, studies showed that patients on GLP-1RA experienced considerable weight loss while on the medication. The mechanism by which GLP-1RA achieves weight loss is by targeting appetite and hunger. GLP-1 is released naturally by the intestines in response to food. GLP-1 receptors are located in the hypothalamus of the brain and upon binding of the GLP-1 peptide, a delay in gastric emptying within the first hour after eating occurs, reducing hunger and increasing satiety (2). Accordingly, multiple studies have provided evidence that GLP-1RA are associated with reduced appetite and a decrease in food cravings. In 2005, the Food and Drug Administration (FDA) initially approved exenatide as the first GLP-1RA for use as an adjunct to diet and exercise to improve glycemic control in those with T2DM. There are now eight GLP-1RA that are approved for use as adjuncts to diet and exercise to improve glycemic control in patients with T2DM. These include dulaglutide once-weekly injection, exenatide once-weekly injection, exenatide twice daily injection, liraglutide once-daily injection, lixisenatide once-daily injection, semaglutide once-weekly injection, oral semaglutide once daily, and newly approved combo therapy Tirzepatide which is a dual gastric inhibitory polypeptide/glucagon-like peptide 1 receptor agonist (GIP/GLP-1RA) (3, 4). With multiple medications on the market and a series of approvals for diabetes and obesity, there has been a sharp rise in the use of GLP-1RA (5, 6). Subsequently, multiple case reports soon emerged showing increased gastric residue in patients on GLP-1RA during EGDs with anecdotal cases of aspiration (7, 8). Following this, an opinion paper in the summer of 2023 published by the American Society of Anesthesiologists (ASA) provided suggestions on preoperative management, such as holding daily GLP-1RA the day of the procedure and holding weekly GLP-1RA a week prior (9). In addition, the ASA suggested delaying EGDs if patients exhibit gastrointestinal symptoms, as there may be a risk of regurgitation and pulmonary aspiration (9). These recommendations resulted in the cancellation of many necessary and scheduled endoscopic and some surgical procedures. As a result, the most recent multi-society clinical practice guidance recommended an individualized approach to preoperative management, which is now the most up-to-date recommendation at the time of this study (10).

Current suggestions to mitigate the risks of aspiration due to GLP-1RA include intubating the patient for airway protection (11). Transabdominal ultrasound is also an option to assess the stomach in patients with symptoms suggesting possible gastric residue, but evidence is still lacking (10). Since a protective effect against retained gastric contents in patients undergoing EGD and colonoscopy has been shown, a liquid diet the day before can be considered (10, 12). In light of the prior guidelines and recommendations to mitigate risks, we aimed to assess food retention in the upper GI tract and the frequency of clinically evident aspiration in consecutive outpatient EGD procedures performed in our tertiary care hospital in order to add to the growing literature.

## Materials and methods

### Study design and patients

This was a retrospective study of 1,368 patients who underwent endoscopic evaluation at an academic University Hospital in Chicago, Illinois. Consecutive, unique EGDs performed on patients in the outpatient setting between September 1, 2022 and October 30, 2023 were reviewed. Our institution's standard pre-procedure instructions state that patients should not consume any food or drink after midnight the day before the procedure. The patients are allowed to take their morning medications with a few sips of water. At check-in for endoscopy, the patients are asked whether they have consumed any food or drink after midnight. Those who have consumed any are rescheduled as, per our institutional guidelines, a fast of 8 h is required for any food other than clear liquids and a fast of 2 h is required for any clear liquids prior to an EGD. This is congruent with the ASA pre-procedural recommendations approved for use prior to endoscopy by the Standards of Practice Committee of the ASGE (11). Patients undergoing same-day colonoscopy are given a split bowel preparation as is standard at our institution, with the first half consumed the evening prior to the procedure. The second half is then consumed on the day of the colonoscopy, about 6 h prior to the scheduled time of the procedure. Adequate preparation is based on the Boston Bowel Preparation Scale (BBPS), with a score ≤ 5 considered inadequate preparation.

### Inclusion and exclusion criteria

**Figure 1:**
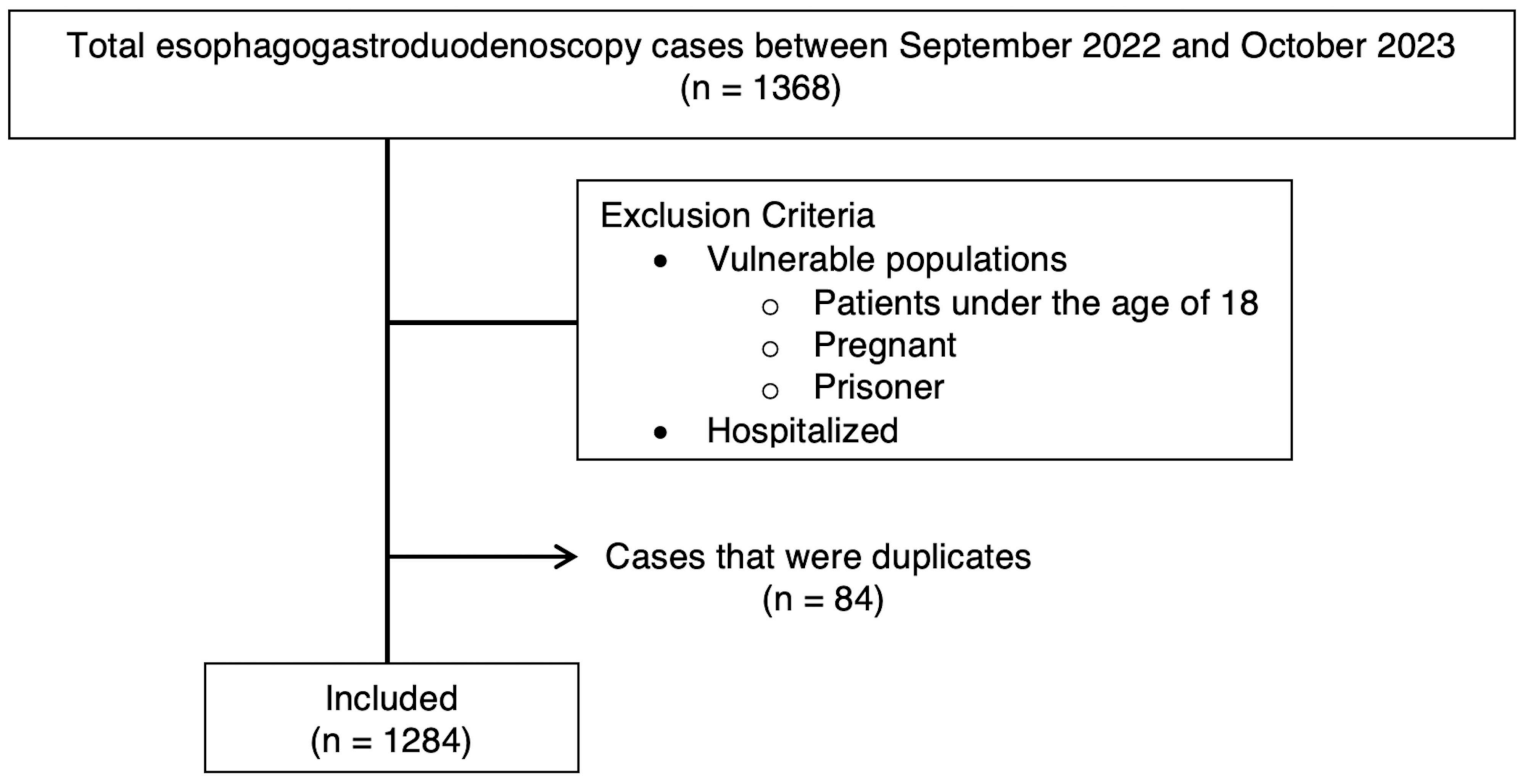
Flow diagram of cases included and excluded from the study.

Inclusion criteria were all EGD procedures performed between the specific dates, and procedure data was extracted from our institution's endoscopy database. Exclusion criteria were patients who were pregnant, incarcerated, under the age of 18, patients with a BMI >50, and patients who were hospitalized. Patients were also excluded if they met the ASA physical status classification of ASA 4 (patients with severe systemic disease threatening life) or ASA 5 (patients who were not expected to survive without surgery) (11). Lastly, each data point represented one unique EGD per person. Therefore, if multiple EGDs were performed on the same individual during this period, only the initial EGD was included (Figure 1).

### Study grouping

Study grouping was defined as GLP-1RA users and non-users. GLP-1RA use was defined as having an active prescription for either semaglutide, dulaglutide, liraglutide, or tirzepatide in the electronic medical record.

### Outcomes and definitions

The primary outcome was retained gastric contents on EGD final report for patients using GLP-1RA (cases) compared to patients not using GLP-1RA (controls). Retained gastric contents were defined as mention of food or fluid in the endoscopy report, which was based on the endoscopist's clinical judgment. Additional information that was extracted included whether the EGD was aborted, which is defined as not achieving a complete evaluation of the esophagus, stomach, and duodenum up to the second portion. Adverse events were defined as any major reduction of oxygen saturation (e.g., requiring reversal medications or unexpected endotracheal intubation), or any other immediate complication (e.g., bleeding, perforation, seizures, or other reasons that required admission of the patient to the hospital following the EGD), as this is standard of clinical care at our institution. Additionally, patients were considered to have an adverse event if they were found to have any late complications (e.g., pneumonia, bleeding, and perforation noted after discharge from the endoscopy lab) as documented during a call from nursing staff occurring seven days after the procedure, which is also standard at our institution. Lastly, patients undergoing same-day EGD-colonoscopy or EGD-flexible sigmoidoscopy were identified as the same cohort because they were all given a bowel prep prior to the procedure.

### Data collection

Data was collected from the electronic medical record on demographics including age, sex, race, and ethnicity. Demographics were defined by patient self-selection for options available in our institution's electronic medical record. Included in these options for both race and ethnicity were unknown/not reported, which was chosen for patients by the researcher for those patients who chose not to select a race or ethnicity. Past medical history, including diagnoses related to the Charlson Comorbidity Index (CCI), and the presence of gastroparesis prior to EGD were also extracted. Lastly, medication history, procedure history, and history of gastric surgery were also extracted as additional risk factors. All collected data was entered into a REDCap database.

### Statistical analysis

The association of the categorical demographic variables for GLP-1RA users vs. non-users was assessed using Fischer's test, which accounted for groups with sample size < 5. A test of normality was used to identify if the continuous demographic variables were normally distributed. Mann-Whitney U Test was used to determine if the distribution of continuous variables was the same across categories of GLP-1RA use. Statistical significance was set at *p* < 0.05. Multicollinearity was analyzed using Spearman correlation to assess the non-parametric data. The correlation coefficient *R* > 0.7 was the cut-off for correlation. A multivariable logistic regression analysis that predicted the outcome of gastric contents was completed. All variables were analyzed together in a single model to evaluate each variable's predictive strength and account for the influence of other variables. The odds ratio (OR) was calculated along with a 95% confidence interval (CI) to assess the strength of the association. The dependent variable, retained gastric contents on EGD, was measured on a dichotomous scale that was mutually exclusive and exhaustive. In order to adjust for confounders, the following covariate variables were selected as the independent variable: age, race, ethnicity, sex, BMI, history of chronic kidney disease (CKD), history of congestive heart failure (CHF), history of diabetes, history of gastric surgery, history of gastroparesis, whether bowel prep was used (e.g., underwent same day EGD-flexible sigmoidoscopy or EGD-colonoscopy), history of GLP-1RA use, and other medications known to contribute to delayed gastric emptying. All data analyses were conducted using SPSS software (version 28.0; IBM Corp, Armonk, NY, USA).

### Power analysis

Using 0.3% baseline rate of adverse events and a dichotomous outcome of occurrence of a complication vs. no occurrence of a complication, at an alpha of 0.05, a sample size of 120 in the GLP-1RA group and a sample size of 1,200 in the control group can detect an increase of 2.7% above the baseline with 80% power (12).

## Results

There were 1,368 patients identified in our study who underwent outpatient EGD. Of these patients, 1,284 met the inclusion criteria, 128 actively used a GLP-1RA, and 1,156 were not using a GLP-1RA. On average, GLP-1RA users had a higher reported BMI and higher age compared to non-users (Table 1). There were more female subjects among the GLP-1RA users. There were also differences in reported GLP-1RA use in different races. For patients identifying as Asian, 73 reported not using GLP-1RA compared to one patient reported as using GLP-1RA, which resulted in a near-complete separation of data for this patient population. For patients reporting as Hawaiian/Pacific Islander, seven reported as not using GLP-1RA and zero reported as using GLP-1RA, resulting in complete separation. There were no other racial differences observed.

**Table 1:** Demographics of study population.

	**GLP-1RA use**	**No GLP-1RA use**	***p*-value**
	**(*****n*** = **128)**	**(*****n*** = **1,156)**	
Mean age	54.55 (SD 11.64)	50.97 (SD 15.31)	0.011
**Sex**			<0.001^***^
Female	101 (78.9%)	728 (63.0%)	
Male	27 (21.1%)	428 (37.0%)	
**Race**			
American Indian/Alaska native	3 (2.3%)	13 (1.1%)	0.209
Asian	1 (0.8%)	73 (6.3%)	0.008^***^
Native Hawaiian or other Pacific Islander	0	7 (0.6%)	0.479
Black or African American	50 (39.1%)	425 (36.8%)	0.63
White	33 (25.8%)	279 (24.1%)	0.665
More than one race	1 (0.7%)	0	0.1
Unknown/not reported	40 (31.3%)	359 (31.1%)	1
**Ethnicity**			
Hispanic or Latinx	53 (41.4%)	423 (36.59%)	0.29
NOT Hispanic or Latinx	72 (56.25%)	712 (61.59%)	0.252
Unknown/not reported	3 (2.34%)	21 (1.82%)	0.726
Mean body mass index	36.72 (SD 6.26)	30.63 (SD 7.76)	<0.001^***^
**Medications**			
Alpha 2 adrenergic agonist	1 (0.8%)	16 (1.38%)	0.571
Calcium channel blocker	17 (13.28%)	221 (19.12%)	0.107
Cyclosporine	0 (0.0%)	7 (0.61%)	0.377
Dopaminergic agonist	0 (0.0%)	4 (0.35%)	0.505
**GLP-1RA**			
Semaglutide (Ozempic)	29 (22.7%)		
Semaglutide (Rybelsus)	8 (6.3%)		
Semaglutide (Wegovy)	11 (8.6%)		
Dulaglutide (Trulicity)	65 (50.8%)		
Liraglutide (Victoza)	14 (10.9)		
Tirzepatide (Mounjaro)	1 (0.8%)		
Immune checkpoint inhibitor	1 (0.78%)	19 (1.64%)	0.455
Muscarinic agonist	4 (3.13%)	37 (3.2%)	0.963
Octreotide	0	3 (0.26%)	0.564
Opiates	8 (6.25%)	73 (6.31%)	0.977
Tricyclic antidepressants	6 (4.69%)	58 (4.98%)	0.871
**Comorbidities**			
Stroke	5 (3.91%)	46 (3.98%)	0.968
Dementia	0	11 (0.95%)	0.268
Hemiplegia	1 (0.78%)	3 (0.26%)	0.315
Connective tissue disease	6 (4.69%)	48 (4.15%)	0.775
Congestive heart failure	10 (7.81%)	28 (2.42%)	<0.001^***^
Diabetes without gastroparesis	90 (70.31%)	221 (19.12%)	<0.001^***^
Diabetes with gastroparesis	5 (3.91%)	13 (1.12%)	0.011^***^
Gastroparesis in non-diabetic	1 (0.78%)	18 (1.56%)	0.49
Chronic kidney disease	13 (10.16%)	34 (2.94%)	0.001^***^
Severe liver disease	3 (2.34%)	45 (3.89%)	0.381
History of gastric surgery	11 (8.59%)	77 (6.661%)	0.412
Charlson comorbidity index	3.4766 (SD 2.792)	2.2690 (SD 2.399)	<0.001^***^
Bowel prep given	43 (33.59%)	469 (40.57%)	0.089

^***^p-value indicates a statistically significant value when < 0.05.

There were 33 patients who were not diabetic and were using a GLP-1RA in our cohort. There were 90 patients with both diabetes and gastroparesis in the GLP-1RA user group. In comparison, there were five patients with diabetes without gastroparesis amongst the GLP-1RA users (Table 1). The CCI was higher among users of GLP-1RA.

### Patients with retained gastric contents

**Figure 2:**
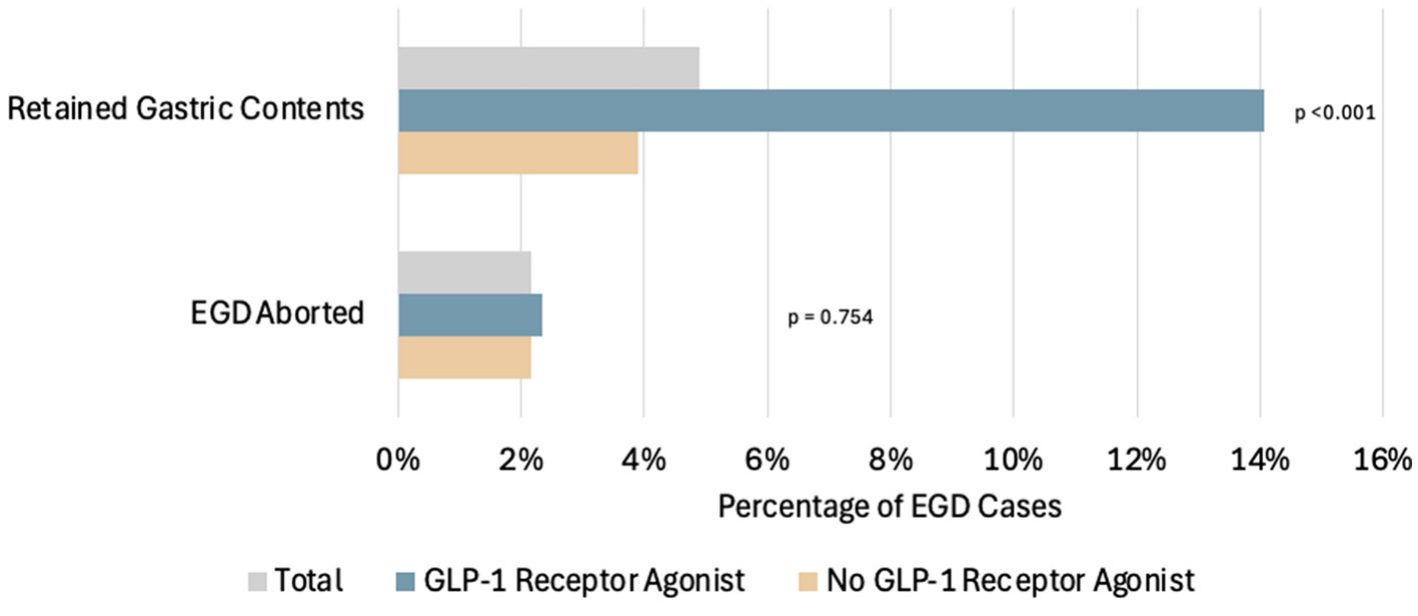
Comparison of the percentage of patients with EGDs with retained gastric contents and the percentage of EGD cases that were aborted in total, among patients using GLP-1RA and among patients not using GLP-1RA. Of the total EGD cases, 63/1,284 (4.91%) had retained gastric contents on EGD. Among the GLP-1RA users: 18/128 (14.1%) had retained gastric contents on EGD, and 3/128 (2.3%) had EGD cases aborted. Among the non-users: 45/1,156 (3.9%) had retained gastric contents on EGD, and 25/1,156 (2.2%) had EGD cases aborted.

**Figure 3:**
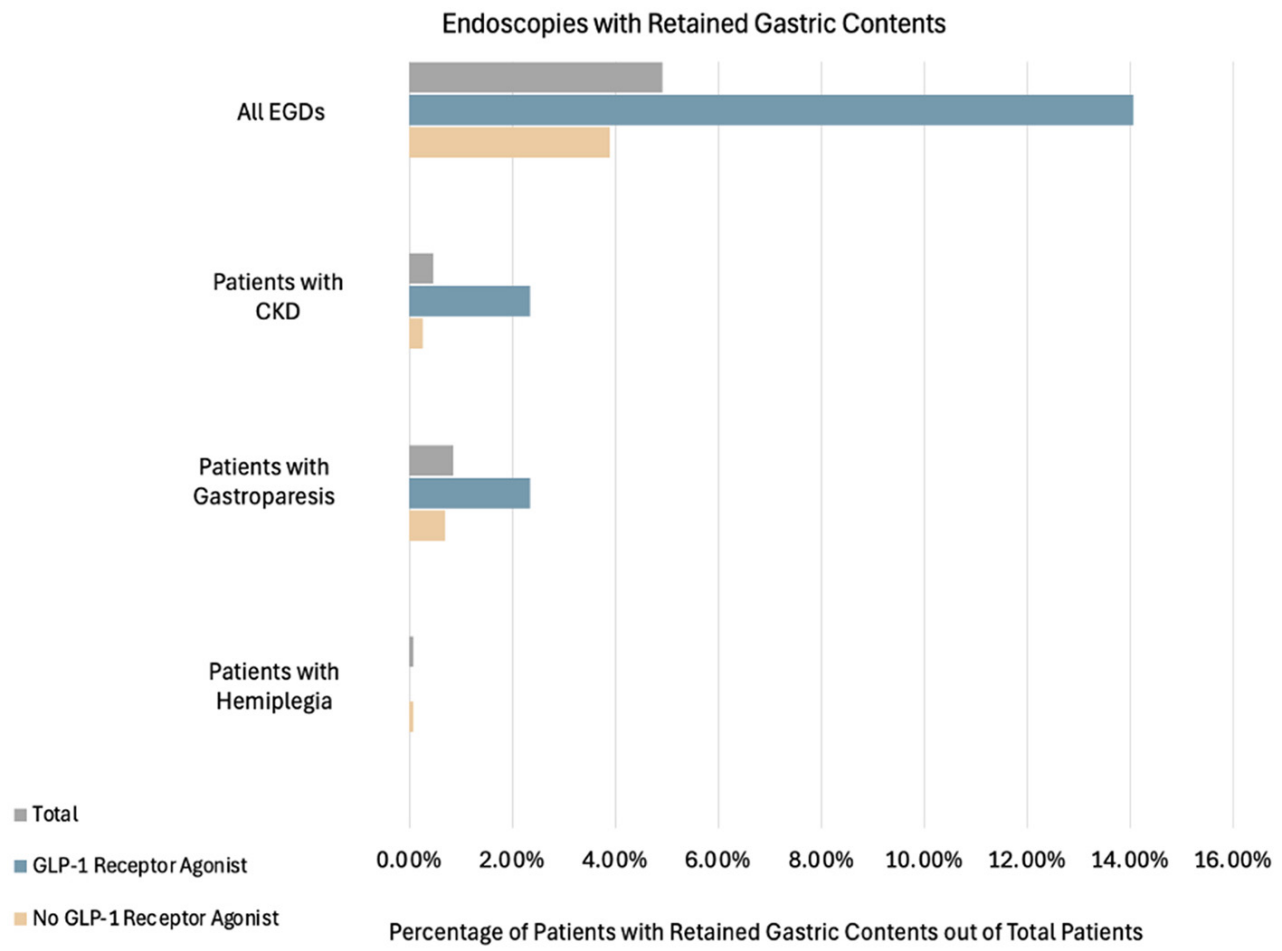
Percentage of patients with retained gastric contents grouped by total patients, GLP-1RA users, and non-GLP-1RA users. Patients who had retained gastric contents and also had either gastroparesis, hemiplegia, CKD, or a combination of any of the aforementioned factors are also shown. Combinations that had outcomes of zero and were not included in the above figure were: gastroparesis+hemiplegia, gastroparesis + CKD, hemiplegia + CKD and all combinations of three or more factors. Of the total EGD cases, 63/1,284 (4.91%) had retained gastric contents. Among the GLP-1RA users: 18/128 (14.06%) had retained gastric contents, 3/128 (2.34%) also had CKD, and 3/128 (2.34%) also had gastroparesis. Among the non-users: 45/1,156 (3.89%) had retained gastric contents, 3/1,156 (0.26%) also had CKD, 8/1,156 (0.69%) also had gastroparesis, and 1/1,156 (0.09%) also had hemiplegia.

Retained gastric contents were seen in 18 out of 128 cases in the GLP-1RA users (14.1%), which was statistically significant when compared to 45 out of the 1,156 non-users (3.8%) (*p* < 0.001, LR 18.323) (Figure 2). Of the 18 patients on GLP-1RA with retained gastric contents, one of these patients had an additional history of CKD and one patient had an additional history of hemiplegia (Figure 3). A logistic regression model was built using gastric contents as the outcome. For dependent variables, GLP-1RA use (*p* < 0.001, OR = 5.4, 95% CI 2.451–12.083), history of gastroparesis (*p* < 0.001, OR = 4.55, 95% CI 1.831–11.322), CKD (*p* = 0.036, OR = 3.47, 95% CI 1.084–11.152) and hemiplegia (*p* = 0.048 OR = 2.9, 95% CI 1.021–325.531) were associated with an increased the risk of retained gastric contents. In contrast, BMI (*p* = 0.009, OR = 0.940, 95% CI 0.897–0.985) and bowel prep due to same day lower GI procedure (*p* = < 0.001, OR = 0.157, 95% CI 0.070–0.352) were associated with a decreased risk of retained gastric contents. The model explained 22.5% of the variance (Nagelkerke *R*^2^) in EGDs with retained gastric contents and correctly classified 95.3%. Additional potential confounders that did not reach statistical significance are shown in Table 2.

**Table 2:** Summary of multivariable analysis.

	**Number of patients total, *n* = 1,284, *n* (%)**	**Number of patients with retained gastric contents on EGD, *n* = 63, *n* (%)**	***p-*value**	**Odds ratio**	**95% CI**
Mean age	51.33 (SD 15.02)	53.52 (SD 14.54)	0.603	1.007	0.981–1.034
**Sex**					
Female	828 (65)	33 (52.38)	0.073	0.580	0.320–1.052
**Race**					
American Indian/Alaska native	16 (1.2)	0	0.999	0.000	0.000
Asian	74 (5.8)	1 (1.59)	0.78	0.137	0.015–1.254
Native Hawaiian or other Pacific Islander	7 (0.5)	0	0.999	0.000	0.000
Black or African American	474 (36.9)	21 (33.3)	0.118	0.473	0.185–1.210
White	312 (24)	19 (30.2)	0.409	0.720	0.330–1.570
More than one race	1 (0.078)	0	1.000	0.000	0.000
Unknown/not reported	398 (31)	22 (34.9)			
**Ethnicity**					
Hispanic or Latinx	475 (37)	23 (36.5)	0.819	1.289	0.147–11.298
NOT Hispanic or Latinx	783 (61)	39 (61.9)	0.525	2.062	0.221–19.209
Unknown/not reported	24 (1.9)	1 (1.59)			
Mean body mass index	31.24 (SD 7.84)	29.70 (SD 7.47)	0.009^***^	0.940	0.897–0.985
**Medications**					
Alpha 2 adrenergic agonist	17 (1.3)	2 (3.2)	0.594	1.686	0.247–11.521
Calcium channel blocker	238 (18.5)	15 (23.8)	0.576	1.235	0.590–2.586
Cyclosporine	7 (0.5)	0	0.999	0.000	0.000
Dopaminergic agonist	4 (0.3)	0	0.999	0.000	0.000
GLP-1RA agonist	128 (9.97)	18 (28)	<0.001^***^	5.442	2.451–12.083
Immune checkpoint inhibitor	20 (1.5)	1 (1.6)	0.565	1.902	0.213–16.971
Muscarinic agonist	41(3.2)	1 (1.6)	0.239	0.279	0.033–2.336
Octreotide	3 (0.2)	1 (1.6)	0.088	13.752	0.674–280.495
Opiates	80 (6.2)	5 (7.9)	0.422	0.599	0.171–2.096
Tricyclic antidepressants	64 (4.98)	2 (3.2)	0.558	0.633	0.137–2.920
**Comorbidities**					
Stroke	51 (4)	3 (4.8)	0.768	0.789	0.163–3.813
Dementia	11 (1)	1 (1.6)	0.444	2.349	0.263–20.951
Hemiplegia	4 (0.3)	1 (1.6)	0.048^***^	18.228	1.021–325.531
Connective tissue disease	54 (4)	3 (4.8)	0.929	1.064	0.274–4.134
Congestive heart failure	7 (0.5)	7 (11.1)	0.213	2.043	0.664–6.286
Diabetes	267 (20.8)	24 (38.1)	0.225	1.572	0.757–3.263
Gastroparesis	37 (2.9)	10 (15.9)	0.001^***^	4.553	1.831–11.322
Chronic kidney disease	47 (3.6)	8 (12.7)	0.036^***^	3.477	1.084–11.152
Severe liver disease	48 (3.7)	3 (4.8)	0.419	0.513	0.102–2.588
History of gastric surgery	88 (6.9)	7 (11.1)	0.423	1.451	0.584–3.604
Bowel prep given	512 (39.8)	9 (14.3)	<0.001^***^	0.157	0.070–0.352

^***^p-value indicates a statistically significant value when < 0.05.

### GLP-1RA type and retained gastric contents

There were 128 patients who reported GLP-1RA use. Within the GLP-1RA users, there were seven out of 29 (24.1%) GLP-1RA users who were using semaglutide (Ozempic) and had retained gastric contents on EGD, vs. 11/99 (11.1%) not using Ozempic who had retained gastric contents on EGD (*p* < 0.001). There was one out of 11 (9.1%) GLP-1RA users on semaglutide (Wegovy) who also had retained gastric contents on EGD vs. 17/117 (14.5%) not using semaglutide (Wegovy) with retained gastric contents on EGD (*p* = 0.426). Lastly, there were 10 out of 65 patients (15.4%) using dulaglutide who also had retained gastric contents on EGD vs. 8/63 (12.7%) not using dulaglutide with retained gastric contents on EGD (*p* < 0.001).

### Patients with aborted EGD procedures

A total of 28 cases were aborted. Aborted cases were noted in three out of the 128 cases for the GLP-1RA user group and 25 out of the 1,156 non-user group, which was not statistically significant (*p* = 0.540) (Figure 2). Out of the 28 aborted cases, eight were due to hemodynamic or respiratory instability, and none of those were in the GLP-1RA user group. Twelve were aborted due to food noted in the upper gastrointestinal tract, and four of these patients were taking a GLP-1RA (all patients were taking dulaglutide). The nine remaining aborted cases were due to patient agitation, and none of these patients were using GLP-1RA.

### Effect of having a same-day colonoscopy with bowel prep

**Figure 4:**
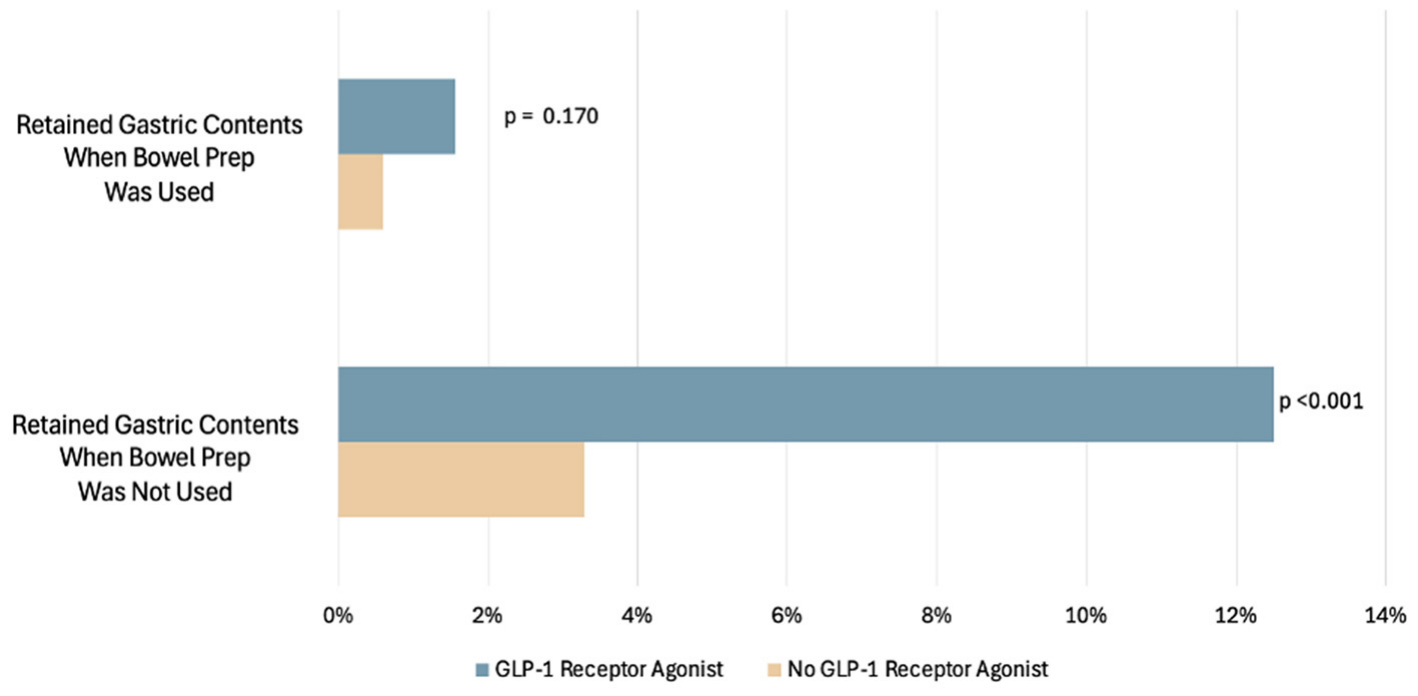
Percentage of patients with EGDs with retained gastric contents compared to those with EGD without retained gastric contents in patients who had bowel prep.

There were 512 total patients who underwent same-day EGD-colonoscopy or EGD-flexible sigmoidoscopy for which bowel prep was used. Fewer GLP-1RA users [43/128 (32.8%)] compared to non-users [469/1,156 (40.6%)] had combined procedures (*p* = 0.016). Of the GLP-1RA users who were given bowel prep, 11/43 (25.6%) had inadequate bowel prep (BBPS ≤ 5). Same-day procedure and bowel prep reduced the percentage of patients with retained gastric contents in both GLP-1RA users and non-users. There were 16/128 (13%) GLP-1RA users with retained gastric contents when bowel prep was not used, vs. 2/128 (2%) with retained gastric contents when bowel prep was used. There were 38/1,156 (3%) non-users with retained gastric contents when bowel prep was not used vs. 7/1,156 (1%) when bowel prep was used (Figure 4). The absolute decrease was 11% for GLP-1RA users, which was statistically significant (*p* < 0.001). Among the patients undergoing same-day procedures who completed a bowel prep, 9/512 (1.76%) had retained gastric contents on EGD. Within the GLP-1RA user group undergoing same-day procedures, 2/43 (4.7%) also had retained gastric contents on EGD, vs. 7/469 (1.5%) within the non-user group (*p* = 0.649). Of the nine patients with same-day procedures, four of the EGD reports indicated food residue as the finding and five of the reports indicated retained fluid as the finding. Lastly, among the patients undergoing same-day procedures, 5/512 (0.98%) had EGD procedures that were aborted, and none of these five cases were in GLP-1RA users.

## Discussion

Our study provides additional support that there is a higher percentage of patients on GLP-1RA with retained gastric contents on EGD. This adds to the findings of the current selection of available studies that show a similar risk of retained gastric contents among users of GLP-1RA. Our study further demonstrates that the risks of significant adverse events such as aspiration are low. Additionally, our study highlights the importance of comorbid conditions that can result in retained gastric contents such as gastroparesis.

When determining the effects of GLP-1RA on endoscopy, it is necessary to look at prior publications in context with our study. Two initial studies showed a significant difference in retained gastric contents, with there being ~5–24% retained gastric contents in GLP-1RA users compared to non-users (13, 14). The first study by Kobori et al. looked at individuals with the diagnosis of diabetes undergoing EGD to see if there were findings of gastric contents in those who used specific GLP-1RA (liraglutide, semaglutide, dulaglutide) compared to those with diabetes who did not use GLP-1RA. The study found that gastric residue was significantly higher in the GLP-1RA user group (14). The second study by Silveira et al. only compared patients taking semaglutide to those not using a GLP-1RA. The study noted that semaglutide use and the presence of ongoing digestive symptoms were significantly associated with increased gastric residue (13). In 2022, Stark et al. retrospectively looked at endpoints of retained food on EGD and found no difference in results for patients using GLP-1RA compared to non-users (95% CI: 0.87–20.34). However, the study was limited to one center and was a relatively small study, with only 135 EGD cases identified (15). Since then, a large meta-analysis of 85 thousand patients across 13 different studies by Facciorusso et al. showed that the use of GLP-1RA is associated with higher odds of increased gastric contents (16). However, the effect of baseline gastroparesis or other comorbidities or use of other medications that could impact gastric emptying independent of GLP-1RA use were not studied. Furthermore, the risk of adverse events and aspiration rates were no different in GLP-1RA users and non-users, similar to our study. A meta-analysis in 2023 tried to assess gastric emptying in patients taking GLP-1RA using various objective measurement modalities. Modalities included scintigraphic measurement of gastric emptying and acetaminophen absorption-based measurement of gastric emptying (17). The analysis had varied results in which the scintigraphic assessment demonstrated that gastric emptying was delayed by 36 min with GLP-1RA use whereas acetaminophen absorption was unaffected by GLP-1RA use (17). While the scintigraphic data indicates a statistical difference, it is not clinically meaningful when it comes to peri-procedural recommendations since patients are already being asked to fast for 8 h prior to any EGD.

In the context of these prior studies, our study adds support for an increase in retained gastric contents for those using GLP-1RA. Notably for our study, there was no significant decrease in endoscopic yields of EGD procedures and no significant increase in adverse outcomes or complications associated with this finding. Thus, the absolute risk of adverse outcomes such as aspiration from an EGD on a patient who has not stopped GLP-1RA prior to the procedure is probably small. Additionally, our study looked at other contributors of retained gastric contents on EGD. There was a significant and independent increased risk for patients with a history of diagnosis of gastroparesis. More recently, although the primary intent was not to study gastroparesis, a multi-center cross-sectional retrospective study by Phan et al. found that patients with diabetes with gastroparesis had a higher gastric content retention rate of 10.8% vs. 3.4% in those without gastroparesis. In the same study the rate of retained gastric contents was also 3.3% in those without diabetes (although only 150 out of 815 patients were not diabetic). Furthermore, increases in hemoglobin A1c heightened the risk of retained gastric contents (18). No aspiration or significant adverse events were noted in those who did not hold their medications despite the fact that the study did not specify whether patients were outpatient/inpatient; and also excluded those having a bowel prep/clear liquid diet the day before the procedure. These findings, along with ours, point to the importance of glucose control in those with diabetes undergoing EGD, and a special need to evaluate those with gastroparesis as well as poor glucose control in conjunction with GLP-1RA use.

In our study, there was also an increased risk for those with hemiplegia and CKD (Figure 3). The risk of delayed gastric emptying in patients with gastroparesis, patients with neurologic deficits, and patients with chronic kidney disease is consistent with the prior literature but has not been previously studied as an independent factor that may increase the risk of retained gastric contents concomitantly with GLP-1RA use (19–23). In our study, the effect of GLP-1RA appeared greater than these underlying comorbidities, with an odds ratio of 5.4. Notably, GLP-1RA use was also a marker of increased comorbidity, with a significantly higher Charlson comorbidity index in those using GLP-1RA in our tertiary center patient population. Our findings suggest that multiple comorbidities, as well as mobility, may need to be taken into consideration before patients are given peri-procedural recommendations regarding GLP-1RA use.

In this study, most patients used dulaglutide and semaglutide (Ozempic). The half-lives of dulaglutide (~90 h) and semaglutide (Ozempic) (~160 h) are substantially longer than some other formulations of GLP-1RAs (24). However, the long-term effect of GLP-1RAs on gastric emptying may not directly correlate with half-life. In two specific studies, the effects of GLP-1RA on gastric emptying diminished with prolonged use, a concept termed tachyphylaxis (25, 26). Of note, our study was not powered to examine any potential differences between short-acting and long-acting GLP-1RAs. Length of exposure to GLP-1RA was also not determined. Our study is also not powered to detect differences in the effects of individual formulations of GLP-1RAs, independent of patient factors. Thus, our subgroup analyses for any individual formulation should be considered exploratory rather than definitive.

Importantly, our results also showed that using bowel prep reduces the risks of retained gastric contents on EGD. In a recently published retrospective study by Nasser et al., GLP-1RA users undergoing EGD were identified and matched to non-users undergoing EGD. They were further stratified into those who underwent EGD alone, EGD-colonoscopy, or colonoscopy alone. The study identified a higher number of patients with retained gastric contents in those undergoing EGD alone compared to no gastric contents in those undergoing combined EGD-colonoscopy (27). Similarly, our study also shows a significant protective effect for those using bowel prep. In our cohort, there were fewer patients in the GLP-1RA user group with retained gastric contents in those who were also given bowel prep (3.3%) compared to those who were not given bowel prep (12.5%). The efficacy of the bowel prep seems to be less in GLP-1RA users. Despite a high percentage of patients with suboptimal prep among GLP-1RA users, the protective effect of bowel prep on retained gastric contents still appears to hold, reducing the rate of retained gastric contents to 3.3% in GLP-1RA users. There is also a similar percentage of those who are not using GLP-1RA in our study, as well as in other studies. It is important to note that the bowel prep did not favor a specific type of retained gastric contents, and patients who had bowel prep had a relatively similar distribution for the type of retained gastric contents of either fluid (identified in four reports) or food (identified in five reports).

It is unclear if the risks are lower with the combination of holding the medication and also having a bowel prep, if one of these approaches may be sufficient to reduce risks, or if even a clear liquid diet without a bowel prep could also be just as effective. In many cases in our outpatient study population, the retained gastric content was fluid and not actual food. Accordingly, measures to mitigate aspiration risk could also include nasogastric tube placement and suctioning of the fluid or gastric contents prior to the procedure, which can be done at the bedside without specialized equipment or additional training. Additional measures could also include intubating selected patients on these medications instead of canceling procedures, since many of these patients also have comorbidities that may necessitate aspiration precautions such as intubation.

Our study has both strengths and limitations. The strengths include that over a thousand consecutive patients were examined, and a comprehensive list of confounders was studied in our dataset. Prior studies have not looked at a comprehensive assessment of comorbidity, such as the CCI. Prior studies also did not examine concomitant medications that delay gastric emptying. Only one study looked at those with gastroparesis. The strengths also include the exclusion of hospitalized patients as well as the inclusion of those with both upper and lower endoscopic procedures to get a comprehensive, real-life assessment of outcomes. The limitations stem from the retrospective nature of the study. The identification of retained gastric contents was not based on a standardized scoring system. Future prospective studies may consider using a validated objective scoring method to standardize the definition of retained gastric contents (e.g., POLPREP) (28–30).

Patient demographic data was also limited to what patients chose to disclose. For example, select patients in the cohort did not identify their race but instead only chose to identify their ethnicity. Other factors such as insurance coverage, cost, availability, and patient preference for administration likely impacted which GLP-1RAs were used. Also, the limited size of certain subgroups of patients impacted results. For example, there was one hemiplegic GLP-1RA user, which resulted in a significant outcome, but with a large confidence interval. Thus, this finding should be considered exploratory. Few patients identified as Asian on GLP-1RA, limiting conclusions for this group. Additionally, while we tried adjusting for confounders in the multivariate analysis model, the list of confounders cannot be considered exhaustive (19–23).

In conclusion, our study adds to the body of literature that GLP-1RA increases retained gastric contents during upper endoscopy. This risk is less for those who have had a same-day colonoscopy, have been on a day of clear liquids, and have taken a bowel prep, mitigating the risk. It is unclear if it is the bowel prep or the clear liquid diet that mitigates the risk. In practice, many EGDs are being canceled for those patients taking their GLP-1RA despite instructions to hold the medications at institutions across the US. This has led to a waste of time, loss of resources, and delayed diagnoses for patients. There is thus an urgent need to put in place measures to prevent the cancellation of procedures while not compromising patient safety. Our findings suggest that EGDs can be safely completed in a majority of patients on GLP-1RA despite not stopping the GLP-1RA medications. Larger studies would be beneficial to validate safety. Additionally, our study suggests a measured approach for patients who have additional comorbidities such as CKD, immobility or a history of gastroparesis. Further studies can determine whether pulmonary complications such as aspiration are significantly higher with GLP-1RA use and determine the exact magnitude of risk, which appears small based on the current available data. Future studies could investigate an extended clear liquid diet period (perhaps 24 h prior to an EGD procedure, similar to what colonoscopy patients currently are asked to do) and an extended nothing-by-mouth period as potential new measures to reduce the risk of retained gastric contents for patients on GLP-1RA.

## Author disclosure

Study data was collected and managed using REDCap electronic data capture tools hosted at the University of Illinois Chicago. 1,2 REDCap (Research Electronic Data Capture) is a secure, web-based software platform designed to support data capture for research studies, providing (1) an intuitive interface for validated data capture, (2) audit trails for tracking data manipulation and export procedures; (3) automated export procedures for seamless data downloads to common statistical packages; and (4) procedures for data integration and interoperability with external sources.

## Data Availability

The original contributions presented in the study are included in the article/supplementary material, further inquiries can be directed to the corresponding author.
